# International Group for Research in the Immunopathogenesis of Genital Infections: II. International Symposium on Immunopathogenesis of Pregnancy, December 6–7, 1997

**DOI:** 10.1155/S1064744997000136

**Published:** 1997

**Authors:** Steven S. Witkin, Myriam Askienazy-Elbhar, Charles Brami, William J. Ledger

**Affiliations:** 1 New York USA; 2 Paris France


					Infectious Diseases in Obstetrics and Gynecology 5:69-72 (I 997)
(C) 1997 Wiley-Liss, Inc.
International Group for Research
in the Immunopathogenesis of
Genital Infections
II. International Symposium on
Immunopathogenesis of Pregnancy
December 6-7, 1997
American Hospital of Paris
63, boulevard Victor-Hugo
92200 Neuilly-sur-Seine, France
Scientific Program Committee
Steven S. Witkin, PhD, New York, USA
Myriam Askienazy-Elbhar, PhD, Paris, France
Charles Brami, MD, Paris, France
William J. Ledger, MD, New York, USA
Sponsors
American Hospital of Paris
Department of Obstetrics and Gynecology, Cornell University Medical College
Pfizer Inc.
BMD (BioMedical Diagnostics), France
Diamant-Roussel-Hoechst, France
Ipsen-Biotech, France
Pharmacia Upjohn, France
CIS-BIO International
Ortho Clinical Diagnostics, France
Stago Diagnostics, France
Biochem, France
Products Roche, France
Program
INTERNATIONAL SYMPOSIUM: IMMUNOPATHOGENESIS OF PREGNANCY PROGRAM
SYMPOSIUM SCHEDULE
SATURDAY, DECEMBER 6, 1997
9:00-9:15 Welcome: Dr. G. Stoleru
SESSION I. BASIC MECHANISMS
William J. Ledger, Chairperson
9:15-9:45
9:45-10:05
10:05-10:15
10:15-10:30
10:30-11:00
Immunopathology of Early Pregnancy
G. Chaouat, Clamart, France
Molecular and Immune Mechanisms of Implantation
P. Fenichel, Nice, France
Implantation in Women Undergoing IVF
C. Brami, Paris, France
Endometriosis: Analysis of 1417 Consecutive Cycles of IVF
S. Spandorfer, New York, USA
Development of the Immune System
A. Durandy, Paris, France
11:00-11:30 COFFEE BREAK AND POSTER VIEWING
11:30-12:00
12:00-12:15
12:15-12:30
12:30-12:45
12:45-13:00
Molecular Mechanisms of Parturition
F. Ferr6, Paris, France
Decidualization Related Expression of Plasminogen Activators in Human Endometrial Stromal
Cells
C. Papp, Budapest, Hungary
Monoclonal Antibody Against Phosphatidylserine Interferes With the Differentiation of Cho-
riocarcinoma Models of Trophoblast
N. S. Rote. Dayton, Ohio, USA
Production of Signal Transducers Differs Between Cultured Cervical and Myometrial Cells
Incubated With Prostaglandin E2
V. Lejeune, Paris, France
Free Fatty Acids and Alpha-Fetoprotein Profiles in the Human Maternal-Fetal-Placenta Unit at
Term Pregnancy
C. Benassayag, Paris, France
13:00-14:00 LUNCH
SESSION II. INFECTIONS
Jeanine Henry-Suchet, Chairperson
14:00-14:30
14:30-14:45
14:45-15:15
Pathogenesis to Treatment: Preventing Preterm Birth Mediated by Infection
J. McGregor, Denver, Colorado, USA
Bacterial Vaginosis and Recurrent Abortion
B. Stray-Pederson, Oslo, Norway
Materno-Fetal Transmission of Human Immune Deficiency Virus
A. Schifer, Berlin, Germany
70 INFECTIOUS DISEASES IN OBSTETRICS AND GYNECOLOGY
INTERNATIONAL SYMPOSIUM: IMMUNOPATHOGENESIS OF PREGNANCY PROGRAM
15:15-15:45
15:45-16:15
16:15-16:30
16:30-17:00
17:00-17:15
17:15-17:30
Immunopathogenesis of Toxoplasmosis in Pregnancy
J. Dupouy-Camet, Paris, France
Chlamydia trachomatis Infection, Immunity, and Pregnancy Outcome
S. Witkin, New York, USA
Investigation of Chlamydia trachomatis Specific Antibodies in Mothers With Stillbirth
M. Gencay, Helsinki, Finland
Pathogenesis of HSV and CMV Infections in Pregnancy
M. Askienazy-Elbhar, Paris, France
CMV Serologic Status of Health Workers
M. Vron, Paris, France
Three Cases of Second Trimester Abortion Due to [3-Streptococcus
J. Henry-Suchet, Paris, France
SUNDAY, DECEMBER 7, 1997
SESSION III. IMMUNOPATHOGENESIS
M. Askienazy-Elbhar, Chairperson
9:00-9:30
9:30-10:00
10:00-10:30
Endocrine-Immune Interactions in Pregnant Non-Human Primates with Intrauterine Infection
M. Gravett, Beaverton, Oregon, USA
Immune Sensitization to the 60kD Heat Shock Protein and Pregnancy Outcome
A. Neuer, New York, USA
Biochemical Markers Predictive of Preterm Delivery
S. Inglis, New York, USA
10:30-11:00 COFFEE BREAK AND POSTER VIEWING
11:00-11:15
11:15-11:30
11:30-11:45
11:45-12:00
Ectopic Pregnancy and Chlamydial Hsp60 Serology--Correlation Between Histopathology,
Adhesions and Serologic Responses to 13 Major Epitopes
I. Sziller, New York, USA
Cyclic Nucleotide Phosphodiesterases in Human Myometrium: Potential Targets for Relaxant
and Anti-Inflammatory Agents
M. J. Leroy, Paris, France
Expression of Endogenous Retrovirus (ERV-3) mRNA is Coincident With Intercellular Fusion
in a Choriocarcinoma Model of Trophoblast Differentiation
L. Lin, Dayton, Ohio, USA
Maternal Serum Early Pregnancy Factor in Multiple Pregnancies
Z-Q. Zheng, Talyuan, People's Republic of China
12:00-13:45 LUNCH
SESSION IV. TREATMENT
J. McGregor, Chairperson
13:45-14:15
14:15-14:45
Prevention of Infection in Pregnancy
W. Ledger, New York, USA
Prevention of Maternal-Fetal Transmission of HIV
J. F. Delfraissy
INFECTIOUS DISEASES IN OBSTETRICS AND GYNECOLOGY 71
INTERNATIONAL SYMPOSIUM: IMMUNOPATHOGENESIS OF PREGNANCY PROGRAM
14:45-15:15
15:15-15:45
15:45-16:00
16:00-16:15
16:15-16:45
16:45-17:05
Cell Damage at the Origin of Antiphospholipid Antibodies and Their Pathogenic Potential in
Recurrent Pregnancy Loss
V. Piroux, V. Eschwge, J. M. Freyssinet, Strasbourg, France
Laboratory Diagnosis of Antiphospholipid Antibodies
M. Samama, Paris, France
Recurrent Fetal Loss and Antiphospholipid Antibodies: Clinical and Therapeutics Aspects
O. B16try, Suresnes, France; S. Uzan, Paris, France
Immunological Studies and Treatment of Women With Unexplained Recurrent Spontaneous
Abortions
R. Georgieva, Sofia, Bulgaria
Screening and Follow-up of Maternal Lymphocyte Cytotoxicity (LCT) and Successful Low
Dose Salicylate (LCS) Treatment of Habitual Aborters During Pregnancy
P. Varga, P6cs, Hungary
Ethical Dimensions of Human Immunodeficiency Virus Infection During Pregnancy
F. Chervenak, New York, USA
Human Immunodeficiency Virus Infections and Reproductive Health: A French Legal and
Ethical Approach
C. Sureau, Paris, France
SESSION V. TREATMENT CONTROVERSIES
M..Gravett, C. Tibi, Chairmen
17:05-17:30 Round Table Discussion
POSTER SESSION
1. Blockage of Implantation in the Rat by a Monoclonal Antibody (MAG)
B. Nemeth, New Haven, Connecticut, USA
2. The Immunology of a Fetus With The Chronic Pyelonephristis of Pregnant Women
T. S. Bystritskaya, Blagoveshchensk, Russia
3. Non-Specific Immunoprophylaxis of the Intrauterine Infection in Pregnant Women with Chronic
Pyelonephritis
T. A. Melachova, Blagoveshchensk, Russia
4. The Immunological Criteria fo Preclinical Forms of Gestoses in Adolescents Primiparous
T. Z. Kozayeva, Blagoveshchensk, Russia
72 INFECTIOUS DISEASES IN OBSTETRICS AND GYNECOLOGY

				

